# A Serious Game for Enhancing Rescue Reasoning Skills in Tactical Combat Casualty Care: Development and Deployment Study

**DOI:** 10.2196/50817

**Published:** 2024-08-12

**Authors:** Siyue Zhu, Zenan Li, Ying Sun, Linghui Kong, Ming Yin, Qinge Yong, Yuan Gao

**Affiliations:** 1 Medical School of Chinese People's Liberation Army, Department of Emergency Medicine the Second Medical Center & National Clinical Research Center for Geriatric Diseases Chinese People's Liberation Army General Hospital Beijing China; 2 Garrison Veteran Cadres Activity Center Beijing China; 3 Department of Emergency Medicine the Third Medical Center Chinese People's Liberation Army General Hospital Beijing China; 4 Health Service Training Center Chinese People's Liberation Army General Hospital Beijing China; 5 Department of Emergency Medicine the Second Medical Center & National Clinical Research Center for Geriatric Diseases Chinese People's Liberation Army General Hospital Beijing China; 6 Department of Nursing the Second Medical Center & National Clinical Research Center for Geriatric Diseases Chinese People's Liberation Army General Hospital Beijing China; 7 Department of Nursing the First Medical Center Chinese People's Liberation Army General Hospital Beijing China

**Keywords:** combat casualty care, simulation training, medical service support, virtual reality, military exercise, medical education

## Abstract

**Background:**

Serious games (SGs) have emerged as engaging and instructional digital simulation tools that are increasingly being used for military medical training. SGs are often compared with traditional media in terms of learning outcomes, but it remains unclear which of the 2 options is more efficient and better accepted in the process of knowledge acquisition.

**Objective:**

This study aimed to create and test a scenario-based system suitable for enhancing rescue reasoning skills in tactical combat casualty care.

**Methods:**

To evaluate the effectiveness of the SGs, a randomized, observational, comparative trial was conducted. A total of 148 members from mobile medical logistics teams were recruited for training. Pre- and posttraining assessments were conducted using 2 different formats: a video-based online course (n=78) and a game simulation (n=70). We designed 3 evaluation instruments based on the first 2 levels of the Kirkpatrick model (reaction and learning) to measure trainees’ satisfaction, knowledge proficiency, and self-confidence.

**Results:**

There were 4 elements that made up the learning path for the SGs: microcourses (video-based online courses), self-test, game simulation, and record query. The knowledge test scores in both groups were significantly higher after the intervention (*t*_154_=–6.010 and *t*_138_=–7.867, respectively; *P*<.001). For 5 simulation cases, the average operation time was 13.6 (SD 3.3) minutes, and the average case score was 279.0 (SD 57.6) points (from a possible total of 500 points), with a score rate of only 44% (222/500 points) to 67% (336/500 points). The results indicated no significant difference in trainees’ satisfaction between the 2 training methods (*P*=.04). However, the game simulation method outperformed the video-based online course in terms of learning proficiency (*t*_146_=–2.324, *P*=.02) and self-perception (*t*_146_=–5.492, *P*<.001).

**Conclusions:**

Despite the high satisfaction reported by trainees for both training methods, the game simulation approach demonstrated superior efficiency and acceptance in terms of knowledge acquisition, self-perception, and overall performance. The developed SG holds significant potential as an essential assessment tool for evaluating frontline rescue skills and rescue reasoning in mobile medical logistics teams.

## Introduction

Serious games (SGs) are video games developed specifically to have an educational purpose [1] and can be used to train both technical and nontechnical skills [2,3]. SGs are defined as representative of nonimmersive systems that have a virtual environment accessed through a display and interactions limited to a keyboard and mouse [3]. SGs have become a useful training technology to learn about health care procedures and a perfect channel to promote learning content [4]. First aid, triage, and mass emergency are the most popular fields taking advantage of the safe and controlled environment [5] provided by virtual reality (VR), wherein games have been developed for training medical doctors or students. Prominent examples include the French Military Health Service’s SG to train for and assess forward combat casualty care (3D-SC1, 2014) [6], the US Army’s tactical combat casualty care simulation training program (TC3Sim, 2020) [7], and the Joint Theater Level Simulation (JTLS 2017) software [8], all of which integrate VR and remote instruction. These software applications furnish soldiers with immersive and repeatable learning experiences, reducing training costs and shortening training periods. As a result, these SGs hold tremendous value as military training applications.

For a long time, the medical service forces at Chinese military hospitals have had limited opportunities to practice combat casualty care and develop the specialized skills required for actual combat situations. This has exposed various issues, including the common misconception of focusing solely on skill practice without adequately addressing decision-making. Working in emergency medicine requires situational assessment and decision-making as well as initiation of appropriate emergency measures under time pressure, often under adverse external conditions and, at the same time, with little or no fault tolerance [9].

To date, published data about SG use in military medical training are limited. Experimental studies often compared learning outcomes in SGs with traditional media, but it remains unclear which of the 2 options is more efficient and better accepted in the process of knowledge acquisition [10]. Some studies have shown SGs’ superiority in specific variables related to learning or training effectiveness [9,11], while others have failed to find a statistically significant difference between these 2 training approaches in terms of learning effects [12,13]. In the study by Hu et al [14], compared with online lectures, the game-based learning approach clearly resulted in better acquisition and retention of information related to COVID-19. Similar studies [15] have also found satisfaction and motivation were greater with SGs than with traditional teaching methods. Several studies have compared the 2 methods but were characterized by a high level of heterogeneity [16] and sometimes provided neutral results [17]. Therefore, it is necessary to compare the 2 training methods. In addition, few articles reported the development process for game development [18].

Therefore, this study aimed to develop an SG and assess its impact, compared with that of a video-based online course, on the learning outcomes of members within mobile medical logistics teams. This innovative approach endeavored not only to provide a new training tool for mass casualty care but also to implement and analyze the practical application of SGs, thereby illustrating their training effectiveness and educational value.

By conducting a comparative evaluation of the video-based online course and game simulation through the constructed SG, the primary goal of this study was to enhance clinical reasoning and procedural reasoning abilities [19]. The intermediate goal was to improve the overall capacity for rescuing combat casualties, while the ultimate goal was to foster the sharing of health training resources and provide support for the rapid and effective development of mobile medical service units.

## Methods

### Ethical Considerations

This study obtained ethics approval from the Institutional Review Board of Chinese People’s Liberation Army (PLA) General Hospital (S2021-043-01). Informed consent was obtained from all participants.

### Design and Development of the SG

#### Phase 1: Software Architecture and Learning Path Composition

Due to the high capacity to create photorealistic environments and visual scripting systems [20], Unreal Engine was used to develop the visual interface scene display and operation of the SG. The system leveraged the Java programming language and a MySQL database to implement the background logic and data recording aspects.

The research group consisted of 1 professor, 1 doctor, and 2 individuals with master’s degrees. The development of the SG used the Kolb Experiential Learning Cycle framework, which achieves effective learning through a cycle of 4 stages: (1) having an experience (“concrete experience”), (2) reflecting on the experience (“reflective observation”), (3) learning from the experience (“abstract conceptualization”), and (4) trying out what you have learned (“active experimentation”) [21]. During case preparation, the research group determined the injuries of the wounded, including injury type, injury position, different treatments, and further course, in each case according to the literature. For example, the Case 3 scenario, “Israeli-Palestinian Conflict,” was designed to be a large-scale air raid, and paramedics needed to rescue 3 wounded personnel with eye trauma, open pneumothorax, and detonation injuries (“concrete experience” and “reflective observation” from the Kolb framework).

During a subsequent Delphi expert consultation, the case script and standard rescue flow chart were sent to 20 experts, and the final version was finalized through 2 rounds of consultation and feedback. Furthermore, participants were given a short presentation about the cases and a review of relevant rescue skills by the teachers. Therefore, the participants learned about the cases experienced in the SG and gained further specific, structured knowledge and skills (“abstract conceptualization” from the Kolb framework).

In a simulation training session, the participants then performed video learning and game operations. The scores of the participants were determined by the software in real time. If desired, participants could also view their scores and errors themselves in the record query. By means of this simulation session, the participants can immediately transfer the newly gained insights to a practice situation such as military exercises and training (“active experimentation” in the Kolb framework).

Finally, the participants reflected in the upcoming rescue mission on the renewed experience with similar cases in a case-based matter. (“concrete experience” and “reflective observation” in the Kolb framework).

There were 4 learning path elements embedded in the SG: microcourses, self-test, game simulation, record query.

The first element, the microcourses (video-based online courses), encompassed 8 video courses that covered the entirety of the knowledge test content. The courses used a short PowerPoint presentation with step-by-step voiceover narration. The learning time was automatically accumulated as the videos were watched.

In the self-test, prior to and after simulation training, participants were required to complete general information forms, knowledge tests, confidence evaluation forms, and satisfaction questionnaires to evaluate the training effects.

In the game simulation, 5 battlefield rescue simulation training scenarios were presented through a combination of tree map options and an answer sheet. The interface displayed a rescue progress bar in the left corner, scrolling to exhibit the specific operations associated with each choice and calculating the current training score. The center area of the interface showed the answer sheet, accompanied by corresponding dialog boxes offering hints and analysis based on the selected rescue measures. Located at the top area were the MARCH principle [22] option buttons representing 5 aspects of casualty care: Massive Hemorrhage, Airway Management, Respiration/Breathing, Circulation, and Hypothermia Prevention. The “Other” options encompassed tasks such as tactical handling, fracture fixation, ocular trauma, burn care, and medical evacuation**.** The timing information was placed in the upper right area, with the final time accessible through the record query.

In the record query, the evaluation record presented the total score, correct rate, and errors. Training records, including lost points, total score, total time, and other data, could be reviewed in the case record.

#### Phase 2: Operation Process

**Figure 1:**
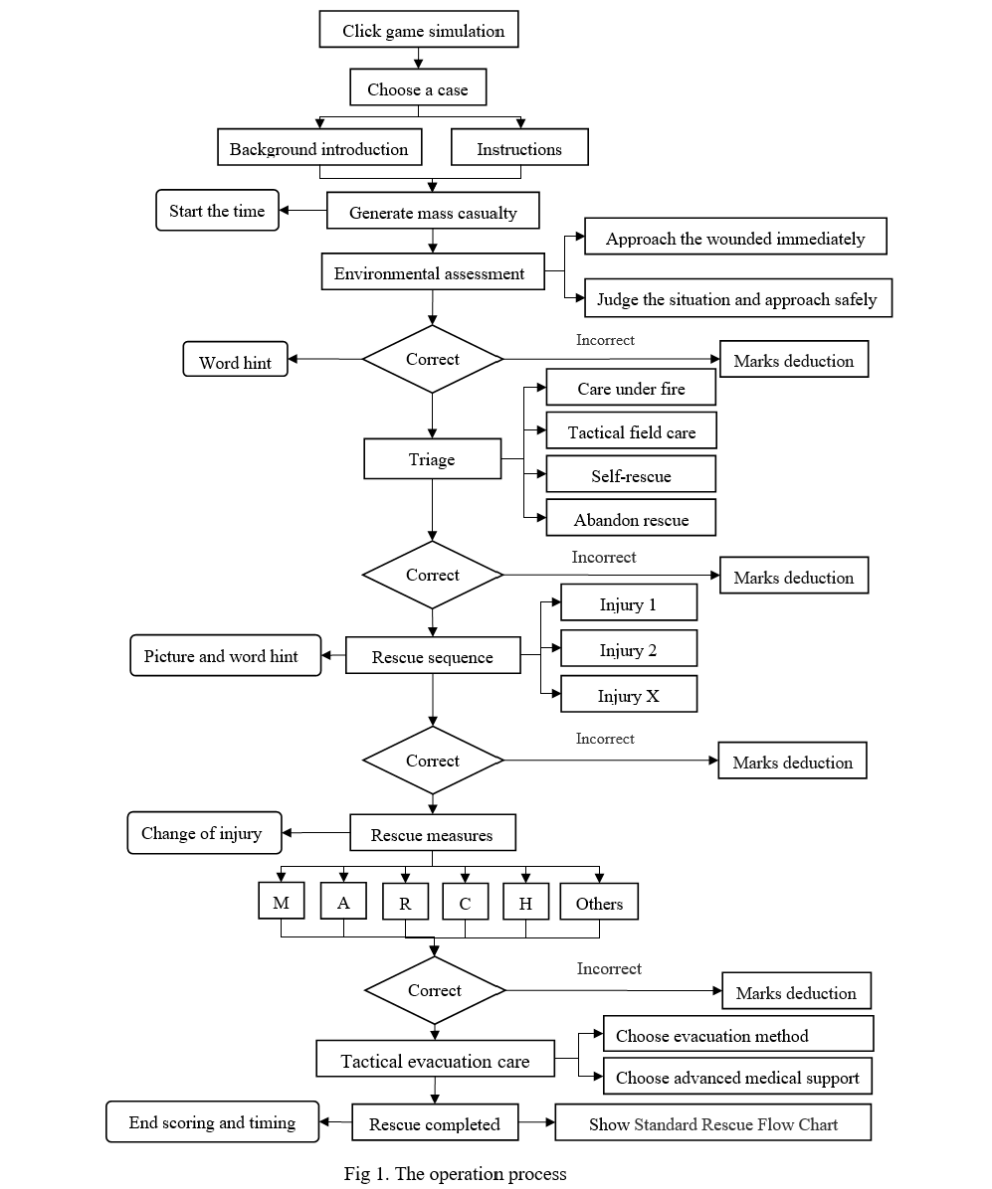
The serious game operation process.

The game simulation was based on the latest tactical combat casualty care (TCCC) guide [22] and divided into 3 stages: care under fire, tactical field care, and tactical evacuation care. Each scenario featured 3 casualties with varying degrees of injury severity. The training focused on injury classification, selection of rescue measures, and evacuation strategies. Notably, upon completion of the rescue, a standard rescue flow chart was automatically displayed, highlighting the correct treatment measures, complete procedures, and the missed procedures for which the automatic virtual instructor had to take control of the experience to perform the procedure [23]. The operation process is shown in Figure 1.

### Deployment Evaluation

#### Design and Sampling

This study was a randomized, observational, comparative study. From January 2023 to March 2023, participants were selected from the mobile medical logistics teams at the Second Medical Center of the PLA General Hospital, the Third Medical Center of the PLA General Hospital, and the Medical Service Training Center using convenience sampling.

The exclusion criterion was that the participant was not a member of the mobile medical logistics team or dropped out halfway.

This research involved software development based on a laptop computer. No headsets nor other related devices were used in this study, so there was no VR syndrome such as dizziness or headache. This study had no impact on human health and safety, so there was no trial registration number.

The required sample size for the comparison of 2 sample means was calculated according to the formula N=[(Z_α/2_+Z_β_)σ/δ]^2^(Q_1_^-1^+Q_2_^-1^) [24]. Based on the differences in mean pretest knowledge test scores between the Control group (microcourses; 9.09, SD 4.944) and the Observation group (game simulation; 11.64, SD 3.392) and considering δ=2.55, σ=4.168, Z_α/2_=1.96, and Z_β_=1.28, the total sample size in both groups was calculated to be 111.51. Considering a sample loss rate of 10%, the total sample size in both groups should be no less than 122, with no fewer than 61 samples in each group. In this study, a total of 155 subjects were initially included, but 7 were lost to follow-up, resulting in final analysis of complete data from 148 participants.

#### Intervention

For randomization, participants were grouped using computer-generated random numbers. A researcher (data processor) who was not informed of the purpose of the study selected the participants in a 1:1 ratio in a sequentially numbered, sealed envelope. After receiving informed consent from each participant, they were randomized by the same researcher. Usually, in web-based trials, it is not possible to blind the participants, but the researcher (outcome assessor) was blinded.

**Figure 2:**
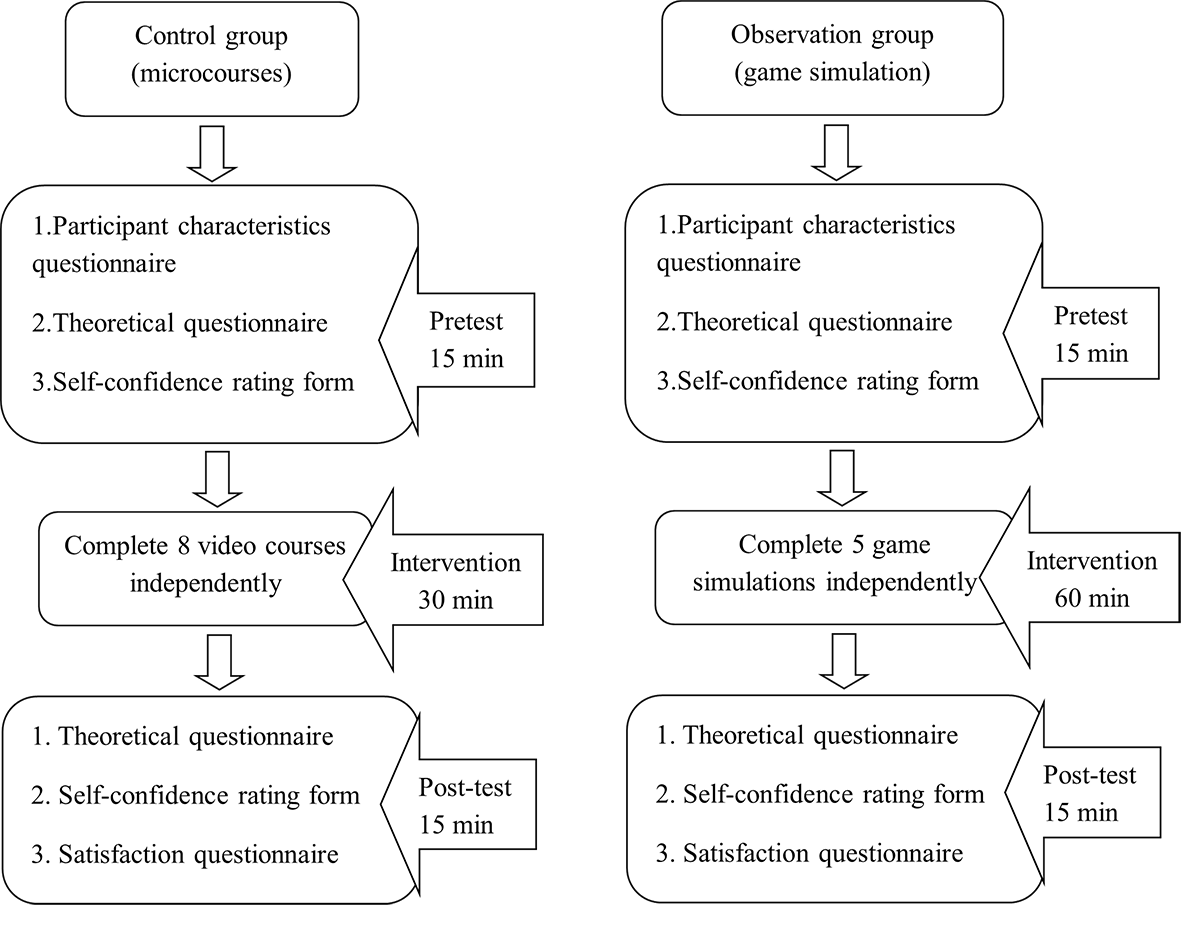
Simulation intervention.

Both groups used the software in a single-player format, with the Control group taking approximately 60 minutes and the Observation group taking approximately 90 minutes. The detailed implementation of the intervention is shown in Figure 2.

#### Instruments

The Kirkpatrick model, a widely used training evaluation model, was used in this study. The model consists of 4 levels: reaction, learning, behavior, and results [25]. Level 3 and level 4 are particularly challenging to observe in an educational learning program [26], as they require postassessment analysis to measure the application of learning in practice and the overall impact on learners. Due to the diverse backgrounds of the research participants involved in this study, it was challenging to assess how effectively learners applied their knowledge in real-time practice and evaluate the overall impact of the learning session. Therefore, evaluation of levels 3 and 4 was not conducted. We developed 3 instruments according to the Kirkpatrick model to assess participant satisfaction and self-confidence (level 1) and the acquired knowledge through pre- and posttests (level 2) to verify the effectiveness and identify shortcomings of the simulation training.

#### Knowledge Test

The questionnaire consisted of 2 parts. The first part assessed participant characteristics, including gender, age, major, education level, years of work, professional title, and previous TCCC training or military exercise participation. The second part was the knowledge test, comprising 25 multiple-choice questions with a total score of 100 points. Each correct answer was assigned 4 points, while an incorrect answer received 0 points. The knowledge questionnaire is available in Multimedia Appendix 1.

#### Self-Confidence Rating Form

This rating form measured participants’ confidence levels in basic knowledge, injury judgment, independent decision-making, and other aspects. Participants were asked to rate their confidence levels on a 5-point scale ranging from “1: Not at all” to “5: Very Much” before and after the simulation training. The self-confidence rating form is available in Multimedia Appendix 2.

#### Satisfaction Questionnaire

The satisfaction questionnaire was based on the simulation training satisfaction scale developed by the National League for Nursing [27] and a previous simulation training research report [28]. It consisted of 10 items related to software interface, method of use, content difficulty, and contribution to comprehensive combat casualty care abilities. A 5-point Likert scale was used for scoring. The satisfaction questionnaire is available in Multimedia Appendix 3.

#### Instrument Validity and Reliability

Using standardized questionnaires helps increase the validity and reliability of the conclusions, as noted by other authors [29].

The content validity of the questionnaires was assessed by 11 military and nursing specialists. Subsequently, the questionnaires underwent a pilot test with a random sample of 45 trainees to assess the software’s feasibility and acceptability, as well as the clarity and understandability of the survey questionnaires. After the pilot test, the 3 evaluation questionnaires demonstrated an acceptable level of content validity (94% agreement for the satisfaction questionnaire, 98% agreement for the knowledge test, and 93% agreement for the self-confidence rating form). In terms of reliability testing, the knowledge test exhibited a retest reliability of 92%, while the satisfaction questionnaire and self-confidence rating form achieved Cronbach α coefficients of 88% and 83%, respectively, indicating good reliability.

### Quality Control

#### Software Application Training

The software training followed the basic principles of simulation-based training [30]: (1) 20 minutes to learn the Operation Manual (briefing and VR familiarization phase); (2) 10-minute demonstration of the location of each functional area and the operation method of the learning path (training phase); (3) 10-minute introduction to the scoring method and discussion of the common problems (training phase); (4) 15-minute explanation of the correct answers and standard rescue procedures, as well as watching case analysis videos (debriefing phase).

#### Data Screening

The research group verified the recovered data and eliminated invalid data. The criteria for invalid data included (1) having only baseline data without postintervention data; (2) omissions exceeding 10% of the total number of questions; (3) answer times significantly less than 50 minutes, which indicated potentially invalid questionnaires; (4) choosing the same option consecutively more than 5 times, which raised concerns about invalidity; (5) unreasonable answers comprising ≥20% of the total answers, which indicated low reliability and invalid data.

### Statistical Analysis

All data analyses were conducted using SPSS version 21.0 (IBM Corp). Continuous data are presented as mean (SD), and categorical variables are presented as counts and percentages. To compare means between the 2 groups, *t* tests were used when the data met the criteria of a normal distribution and homogeneity of variance. The Mann-Whitney rank sum test was used when the data did not meet the criteria of a normal distribution and homogeneity of variance. Chi-square tests were used to analyze categorical data. If the criteria of a normal distribution and homogeneity of variance were met, a 1-way ANOVA was used. When the data distribution was normal but the variance was not, the Dunnett T3 result was selected for multiple comparisons between 2 pairs in the group. If neither a normal distribution nor homogeneity of variance could be assumed, the Friedman rank sum test was used. A *P*<.05 was considered statistically significant.

## Results

### Design and Development of the SG

#### Learning Path Composition

**Figure 3:**
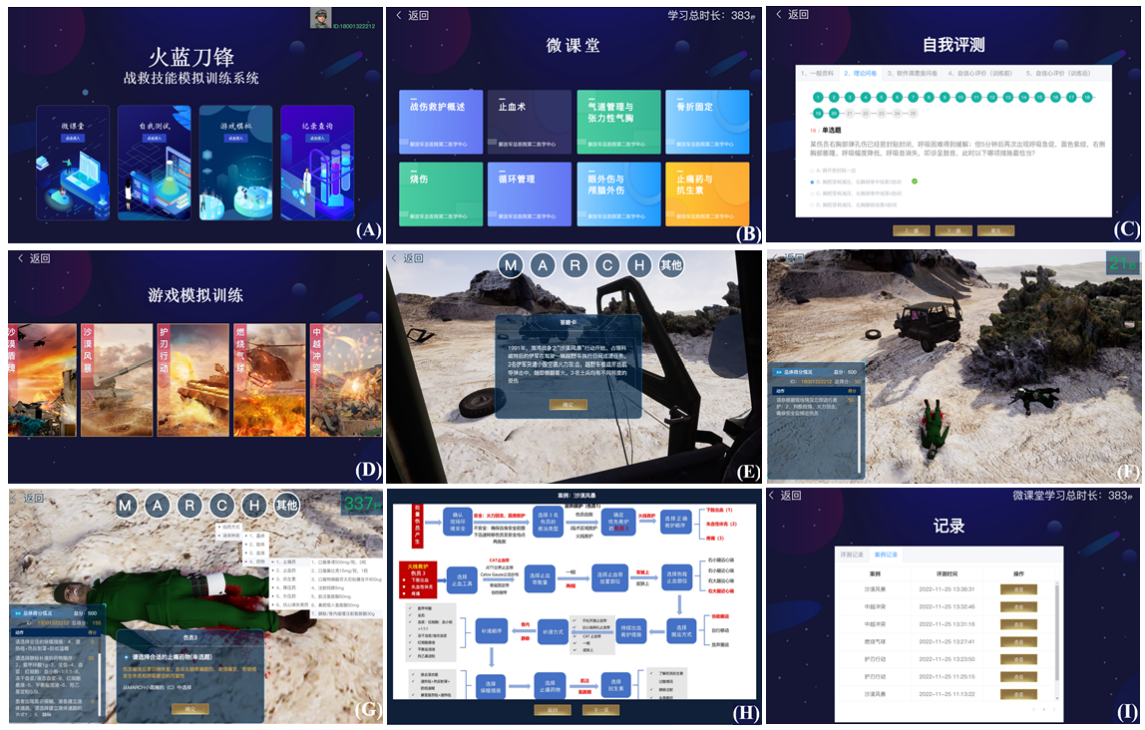
Software interface of the simulation training system for tactical combat casualty care (TCCC) skills, including the (A) home page; (B) microcourses: TCCC summary, hemostasis, airway management and tension pneumothorax, fracture fixation, burn treatment, cycle management, eye trauma and craniocerebral trauma, painkillers and antibiotics; (C) self-assessment; (D) game simulation including 5 cases; (E) case background; (F) distant view of the casualty, the total score, the action to take, and score associated with that action; (G) close shot of the wound, with a task to choose the pain medication and the result of the choice; (H) standard rescue flow chart using patient 3 as the example; (I) record query including the evaluation records and case records.

There are 4 elements that make up the learning path for the SGs: video-based online courses, self-test, game simulation, and record query. See Figure 3 for the interface diagrams of different elements.

#### Game Characteristics

The selection buttons were meticulously designed based on a logical tree graph that followed a sequential and deductive structure. Starting from the MARCH principle as the initial selection point, subsequent branches cascaded downward, clearly delineating the sequence of rescue measures. This approach facilitated understanding and memory retention of the learning content while enhancing the ability to make informed judgments in battlefield rescue situations. Moreover, the overall preview of the tree graph enabled operators to have a comprehensive view of all rescue measures, facilitating a better grasp of the overall rescue layout. The hierarchical structure of the selection buttons ensured an intuitive and efficient operation, aiding in organizing rescue thinking and making prompt rescue decisions.

The case base structure incorporated a compound injury setting, encompassing mass injuries of a battle group and multiple injuries of individual soldiers. The content of injuries was thoughtfully organized to present a reasonable gradient of “first aid + self-rescue,” ensuring a gradual learning effect. By addressing the complex treatment challenges associated with mass casualties, the system transcended the limitations of individual soldier skill training. This comprehensive approach not only honed triage, self-rescue, and mutual rescue abilities but also fostered the accumulation of experience in mass injury treatment among team members, ultimately enhancing overall rescue capabilities on the battlefield.

### Deployment Evaluation With Targeted Users

#### Study Population

Complete data were available from 148 participants: 78 participants in the Control group and 70 participants in the Observation group. The demographic characteristics of the participants were similar between the 2 groups, as shown in Table 1.

**Table 1:** Participant characteristics (n=148).

Variables		Control group (n=78)	Observation group (n=70)	Chi-square (*df*)		*Z* score		*P* value	
**Gender, n (%)**					1.5 (1)		—^a^		.21
	Male	40 (51)	43 (61)						
	Female	38 (49)	27 (39)						
Age (years), mean (Q1-Q3)		30 (28-34)	29 (25-33)	—		–1.655		.10	
**Major, n (%)**					3.3 (2)		—		.20
	Medicine	23 (30)	29 (41)						
	Nursing	35 (45)	30 (43)						
	Logistics	20 (25)	11 (16)						
**Education, n (%)**					0.6 (3)		—		.89
	Diploma	14 (18)	12 (17)						
	Bachelor’s degree	42 (54)	37 (53)						
	Master’s degree	7 (9)	9 (13)						
	Doctorate	15 (19)	12 (17)						
Work duration (years), mean (Q1-Q3)		8 (6-11)	7 (4-10)	—		–1.458		.15	
**Professional title, n (%)**					2.5 (2)		—		.29
	Junior	36 (46)	41 (59)						
	Intermediate	33 (42)	24 (34)						
	Senior	9 (12)	5 (7)						
**Received TCCC^b^ training, n (%)**					3.7 (1)		—		.06
	Yes	71 (91)	56 (80)						
	No	7 (9)	14 (20)						
**Participated in military exercises, n (%)**					0.4 (1)		—		.51
	Yes	52 (67)	43 (61)						
	No	26 (33)	27 (39)						

^a^Not applicable.

^b^TCCC: tactical combat casualty care.

#### Comparison of Indicators Between the 2 Groups

##### Theoretical-Level Comparison of the 2 Groups Before and After the Intervention

The results of intergroup comparisons showed no significant difference in the knowledge test scores between the 2 groups before the intervention (t_146_=1.605, *P*=.11). However, the Observation group had a higher score than the Control group after the intervention (t_146_=–2.324, *P*=.02). In terms of intragroup comparisons, the knowledge test scores in both groups were significantly higher after the intervention (Control group: t_154_=–6.010, *P*<.001; Observation group: t_138_=–7.867, *P*<.001); see Table 2 for details.

**Table 2:** Comparison of the theoretical level of the 2 groups.

Group	Knowledge test score		*t* test (*df*)	*P* value
	Pre-intervention, mean (SD)^a^	Postintervention, mean (SD)^b^		
Control group (n=78)	68.15 (13.47)	78.77 (7.88)	–6.01 (154)	<.001
Observation group (n=70)	64.23 (16.26)	82.20 (10.04)	–7.867 (138)	<.001

^a^Comparison between groups: t_(146)_=1.605, *P*=.11.

^b^Comparison between groups: t_(146)_=–2.324, *P*=.02.

**Figure 4:**
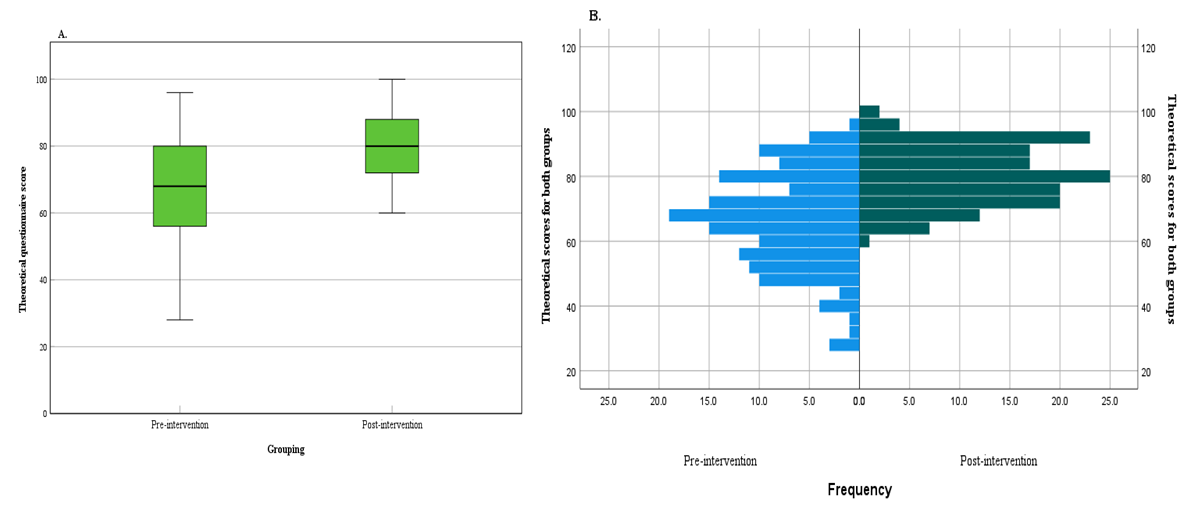
Knowledge test scores: (A) box plots, (B) frequency histogram.

Box plots and frequency histograms of the knowledge test scores for the 148 participants before and after the intervention are shown in Figure 4A and Figure 4B. The average score before the intervention was 66.30 (SD 14.93) points, ranging from 28 points to 96 points. After the intervention, the average score was 80.39 (SD 9.10) points, ranging from 60 points to 100 points. According to the frequency histogram, there was a significant overall improvement in the theoretical level of the participants after the intervention (t_294_=–9.805, *P*<.001).

##### Comparison of the Confidence Levels of the 2 Groups Before and After the Intervention

The results showed significant differences in the scores of 3 items after the intervention: item 4 “I have the confidence to prioritize the injuries” (*P*<.001), item 5 “I have the confidence to discern the changes of injury independently” (*P*<.001), and item 6 “I have the confidence to manage injuries independently” (*P*<.001). However, there was no statistically significant difference in the scores of the other items after the intervention (*P*=.13 to *P*=.34). Refer to Table 3 for more details.

**Table 3:** Average confidence scores compared between the 2 groups after the intervention.

Confidence items	Control group (n=78), mean	Observation group (n=70), mean
I have confidence in my basic knowledge.	3.71	3.49
I have faith in the latest ideas and research progress.	3.26	3.43
I have confidence I can assess the injury accurately.	3.35	3.5
I have the confidence to prioritize the injuries.	3.13	3.83
I have the confidence to independently discern the changes in the injuries.	3.31	3.84
I have confidence to independently manage the injuries.	3.42	4.06
I have the confidence to participate in the military exercises.	4.05	4.19
I have the confidence to fulfill the duties and missions of the mobile medical logistics teams.	4.22	4.33

##### Comparison of Satisfaction Between the 2 Groups After the Intervention

There were statistically significant differences in the scores of 3 items after the intervention: item 2 “Easy to use” (*P*<.001), item 5 “Arouse the learning interest of TCCC” (*P*=.01), and item 9 “Promote the combination of theory and practice” (*P*<.001). However, there was no significant difference in the scores of the other items (*P*=.08 to *P*=.72). The total satisfaction scores did not differ significantly between the 2 groups (*P*=.37). Refer to Table 4 for more details.

**Table 4:** Satisfaction scores of the two groups after the intervention.

Satisfaction items	Control group (n=78), mean	Observation group (n=70), mean
Vivid software interface	3.91	3.87
Easy to use	4.08	3.37
Appropriately difficult injury	4.19	4.14
Prompt, effective answers	4	4.09
Aroused interest in learning TCCC^a^	4.03	4.39
Improved basic TCCC knowledge	4.26	4.37
Increased the importance of TCCC for me	4.1	4.27
Improved my reasoning ability within TCCC	4.1	4.17
Promoted the combined use of theory and practice	3.55	4.03
Ensured the study of TCCC serves a practical purpose	4.23	4.29

^a^TCCC: tactical combat casualty care.

#### Comparison of Indicators in the Observation Group

##### Comparison of Scores and Operation Times Between 5 Cases

**Figure 5:**
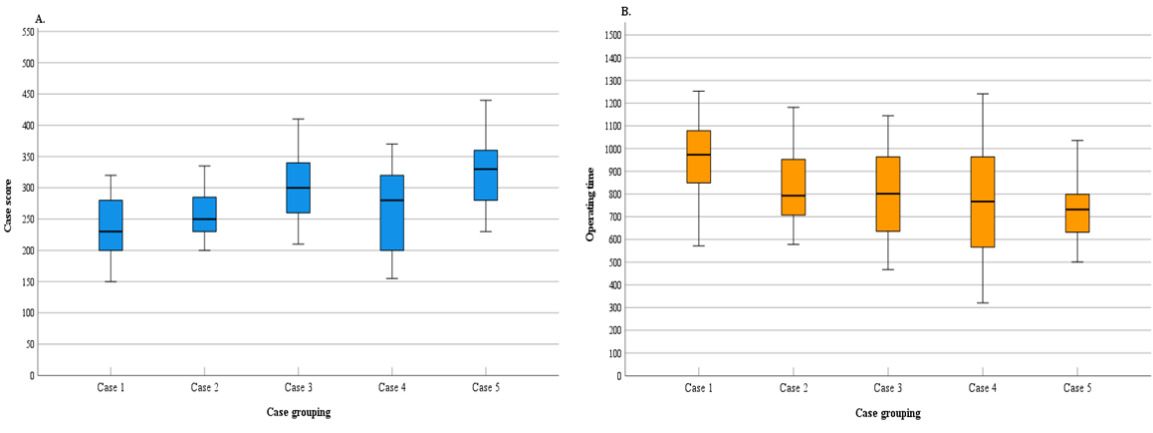
Boxplots of the (A) operating times for the 5 cases and (B) scores of the 5 cases.

The distributions of the case scores (IQR 58.75) and operation times (IQR 179.25) for case 5 were the most concentrated, while the distributions of case scores (IQR 120) and operation times (IQR 405.5) for case 4 were the most dispersed. The box plot charts in Figure 5A and Figure 5B show the distribution of case scores and operation times, respectively, for the 5 cases.

In terms of intergroup comparisons, there were significant differences in the scores for the 5 cases (*P*<.001) and in the operation times for the 5 cases (*P*<.001), indicating an overall increase in case scores and a decrease in operation times with increasing training. The comparisons of case scores and operation times in the Observation group are shown in Table 5.

**Table 5:** Comparison of scores and operation times within the Observation group.

Index	Case 1: Desert Shield, mean (SD)	Case 2: Desert Storm, mean (SD)	Case 3: Sword Guard, mean (SD)	Case 4: Burning Balloon, mean (SD)	Case 5: Conflict of China-Vietnam, mean (SD)	Entire group, mean (SD)	*F* (*df*)	*P* value
Score	240.00 (42.81)	259.29 (36.45)	302.07 (54.09)	270.91 (62.50)	322.79 (48.49)	279.01 (57.69)	31.36 (4,345)	<.001
Operation time (seconds)	967.43 (164.59)	840.83 (176.05)	803.91 (188.60)	765.07 (238.32)	727.50 (134.27)	820.95 (200.32)	17.66 (4,345)	<.001

##### Intragroup Comparison of Case Scores and Operation Times

There were significant differences between the scores of the cases, except for the mean differences between case 2 and case 4 and between case 3 and case 5. See Table 6 for details. There were significant differences in operation times between case 1 and cases 2-5 and between case 2 and case 5 (all *P*<.001; as shown in Table 7).

**Table 6:** Intragroup comparisons of case scores.

Groupings	Mean difference (SE)	*P* value	95% CI
Case 1 - Case 2	–19.286 (6.720)	.05	–38.40 to –0.17
Case 1 - Case 3	–62.071 (8.245)	<.001	–85.53 to –38.61
Case 1 - Case 4	–30.914 (9.054)	.009	–56.71 to –5.12
Case 1 - Case 5	–82.786 (7.731)	<.001	–104.77 to –60.80
Case 2 - Case	–42.786 (7.796)	<.001	–65.00 to –20.58
Case 2 - Case 4	–11.629 (8.648)	.86	–36.30 to 13.05
Case 2 - Case 5	–63.500 (7.250)	<.001	–84.14 to –42.86
Case 3 - Case 4	31.157 (9.879)	.02	3.06 to 59.25
Case 3 - Case 5	–20.714 (8.682)	.17	–45.40 to 3.97
Case 4 - Case 5	–51.871 (9.455)	<.001	–78.77 to –24.97

**Table 7:** Intragroup comparisons of operation times.

Grouping	Mean difference (SE)	*P* value	95% CI
Case 1 - Case 2	126.600 (28.806)	<.001	44.70 to 208.50
Case 1 - Case 3	163.514 (29.919)	<.001	78.44 to 248.59
Case 1 - Case 4	202.357 (34.617)	<.001	103.76 to 300.96
Case 1 - Case 5	239.929 (25.388)	<.001	167.71 to 312.15
Case 2 - Case 3	36.914 (30.837)	.93	–50.76 to 124.58
Case 2 - Case 4	75.757 (35.414)	.29	–25.05 to 176.57
Case 2 - Case 5	113.329 (26.463)	<.001	38.02 to 188.64
Case 3 - Case 4	38.843 (36.325)	.96	–64.51 to 142.19
Case 3 - Case 5	76.414 (27.671)	.06	–2.38 to 155.21
Case 4 - Case 5	37.571 (32.694)	.94	–55.76 to 130.90

## Discussion

### Principal Findings

This study established a novel simulation training system that met the need for decision-making in the training field of TCCC. First, Unreal Engine 4 and Virtual University Enterprises were used to develop 5 battlefield rescue simulation training scenarios, and 3D modeling was used to build simulated tactical scenarios. This enabled us to train TCCC skills in a tactical background.

Second, an integrated training mode of “online course, knowledge self-test, game simulation, and error review” was constructed, which provided a new training method to improve decision-making ability.

Comparison of the performance of the video teaching and game simulation showed that the latter was more effective at improving theoretical knowledge, self-confidence, and comprehensive abilities in TCCC.

### Simulation Training Software: Stimulating Greater Initiative and Cost-Effectiveness

Currently, conducting simulation training for TCCC requires significant capital investment and time. Due to the lengthy training cycle of rotating personnel, regular and large-scale centralized training is challenging to implement. Although video teaching can be used for centralized training, it primarily provides passive learning, lacking in-depth professional knowledge and innovative thinking. Trainees may experience lower levels of interest and motivation, even though they may acquire more with a well-designed slide presentation [31]. The use of simulation training software presents an effective solution to address these challenges.

In simulation training software, trainees interact with “virtual wounded” individuals who provide real-time movement, voice, and expression feedback based on different rescue measures. The overall wound situation is presented through panoramic character shots and close-up shots of specific wounds. By simulating various rescue scenes and conducting detailed examinations of injuries, the software creates a 3D, immersive, interactive VR environment. This immersive experience and sense of presence have been identified as key factors for enhancing learning rates [32]. The interactive VR experiences [33] associated with immersive sensations and presence overcome the limitations of passive information reception in traditional teaching methods and promote learners’ active thinking and problem-solving abilities.

Although the initial development costs of SGs may be high, the expected benefits in terms of improved patient care and error prevention provide a compelling argument for investing in their development. First, SGs offer low management costs. SGs can assess the retention of procedural skills in a more practical, cost-effective manner. Additionally, SGs can accommodate an unlimited number of students, resulting in cost and time savings related to equipment maintenance and training teachers while maintaining a high level of cost efficiency. Second, SGs have a short development and update cycle. Similar to other e-learning applications, SGs allow the updating of cases and modification of content [2] based on feedback obtained through repeated use. Moreover, SGs enable cost-effective training for a larger population of trainees [34,35], with the flexibility of being available anytime and anywhere. In our study, participants were able to access the SG on their personal computers and tablets.

### The Training Effect of the “Serious Game” Exceeds That of “Video Teaching”

As a teaching tool, evidence has shown VR improves learning outcomes, skill performance, cognitive performance, and knowledge retention [36]. In this study, SGs were more effective at improving trainees’ theoretical knowledge, self-confidence, and comprehensive abilities in TCCC. Therefore, SGs can be considered as the preferred training tool for simulation training.

### Kirkpatrick Phase 1: Evaluation of Reaction

In this study, a software satisfaction questionnaire was used to evaluate the interactive graphical user interface and application effect. The overall satisfaction level was high, with no statistical difference in the total scores between the 2 groups (*P*=.37).

The comparison between the 2 groups revealed that the Control group reported lower scores in “Arouse the learning interest of TCCC” (t_146_=–2.806, *P*=.005) and “Promote the combination of theory and practice” (*P*<.001) than the Observation group. This is likely because the video-based learning mode resembled offline collective teaching [37], lacking interest, interaction, and the effective stimulation of learning enthusiasm, resulting in a significant satisfaction gap between the 2 groups. Only “Easy to use” scored lower in the Observation group than in the Control group (t_146_=–5.977, *P*<.001). This may be attributed to the clear structure of the selection buttons based on the logical thinking tree, allowing operators to preview the overall rescue options. However, the case database contains 5 first-level indicators and 36 second-level indicators, leading to potential confusion for learners with weak logical thinking abilities. Consistent with previous studies [20], trainees who experienced the VR environment reported higher levels of satisfaction. The software successfully enhanced learner enthusiasm and interest by providing well-organized content, appropriate difficulty levels, and valuable feedback, thereby indirectly improving overall learning outcomes.

### Kirkpatrick Phase 2: Evaluation of Learning

The second level of the Kirkpatrick model involves learning assessment. Knowledge tests, self-confidence rating forms, and comparison of scores between the 2 groups were used to assess the level of knowledge and ability acquired by trainees.

#### Knowledge Level: TCCC Knowledge Test

Both groups showed significant improvements in theoretical scores from baseline to after the intervention, indicating a perceived increase in knowledge and clinical judgment, which is consistent with findings from other studies [38,39]. After the intervention, the Observation group achieved higher scores than the Control group (t_146_=–2.324, *P*=.02), suggesting that the SG self-learning method was statistically superior to the video-based self-learning method in terms of knowledge acquisition.

#### Self-Perception Level: Self-Confidence Rating Forms

Except for 3 items, the difference in self-confidence between the 2 groups was small. Although the sense of duty could not be improved through a single training session, the results suggested that software development should focus on injury judgment as the next step. Overall, there was a significant difference in total self-confidence scores between the 2 groups after training (*P*<.001). Intragroup and intergroup comparisons revealed that confidence scores in both groups improved from baseline to after training. Therefore, it can be confidently concluded that training enhances learners’ self-confidence. Research has shown that learners are very motivated to use SGs, because they are more engaging and interactive and provide more continuous feedback than traditional learning methods [40] or e-modules [41]. These results align with previous studies [9] reporting that SG training led to a significant increase in intrinsic motivation among participants.

#### Comparison of Indicators in the Observation Group

When comparing the scores between the 5 cases using box charts and histograms, the trainees’ mastery of rescue measures for massive hemorrhage, hemorrhagic shock, and closed fractures was consistently high, while mastery of burns and traumatic brain injury showed more variability. Although there was no significant difference in scores between the 2 cases (*P*=.86), the scores for traumatic brain injury fluctuated greatly, indicating a poor understanding of these knowledge points and the need for consolidation and intensive training to minimize differentiation and internalize knowledge.

#### Intragroup Comparisons

Regarding intragroup comparisons, the results suggested that the treatment level for tension pneumothorax (Case 1) was not as good as for other injuries. Tension pneumothorax is a special form of open pneumothorax with insidious clinical manifestations and no obvious symptoms other than dyspnea, leading to misjudgment. This indicates the need for further improvement in the trainees’ recognition of injuries and clinical logical reasoning skills.

The average operation time for the SG was 13.68 (SD 3.34) minutes, and the average case score was 279.01 (SD 57.69) points. Previous studies reported average operation times ranging from 195.09 (SD 72.03) to 350.00 (SD 108.36) seconds or 17 minutes to 25 minutes within training scenarios [42]. Therefore, the operation time in our study was deemed reasonable, ensuring adequate attention to each case. The average case score differed significantly from the full score of 500, with a score rate of only 44% (222/500 points) to 67% (336/500 points). These results indicate that the members of the mobile medical service team did not have a good grasp of TCCC knowledge, necessitating reteaching and retraining on the fundamentals of TCCC.

### Conclusions

This study developed an innovative tool for TCCC training, complementing offline practical operations. It enhanced overall training effectiveness and provided a new method for training mobile medical logistics teams. The results showed that the “game simulation” achieved better training effects than the “microcourses” for theoretical knowledge, self-confidence, and comprehensive abilities.

The software successfully stimulated the learning enthusiasm and interest of trainees by providing well-organized content, appropriate difficulty levels, and valuable feedback. It improved the trainees’ knowledge levels, self-perception levels, and overall performance. The software enabled cost-effective training for a larger population of trainees, with the flexibility of being available anytime and anywhere.

However, the software still requires improvement in terms of wounded model development, wound spectrum representation, rescue equipment, and other aspects. Future research will focus on capturing behavioral changes and resulting benefits among learners. Further investigation will also involve multicenter verification, the exploration of additional human-computer interaction methods, and the creation of more natural and engaging intelligent user interfaces. These efforts aim to align with emerging trends and attract more young officers and soldiers to participate in TCCC simulation training.

The limitations of this study include the convenience sampling method, lack of long-term follow-up data, limited comparable context for the applied VR technology [42], hardware and software constraints, current deployment only on PCs and laptops, and the need to establish an extensive database.

## Appendix

Multimedia Appendix 1 Knowledge test.

Multimedia Appendix 2 Self-confidence rating form.

Multimedia Appendix 3 Satisfaction questionnaire.

## Abbreviations

JTLS: Joint Theater Level Simulation
PLA: People’s Liberation Army
SG: serious game
TCCC: tactical combat casualty care
VR: virtual reality

## Data Availability

The data sets generated during and/or analyzed during this study are available from the corresponding author (YG) on reasonable request
