# Supplementary material for: A Serious Game for Enhancing Rescue Reasoning Skills in Tactical Combat Casualty Care: Development and Deployment Study
Source: JMIR Form Res. 2024 Aug 12;8:e50817. doi: 10.2196/50817 (PMC11347892; doi:10.2196/50817)
Supplement: Multimedia Appendix 1 [file formative_v8i1e50817_app1.doc]

**Appendix 1**

**Knowledge Test**

**Part I General information**

1. Gender: A. Male B. female

2. Age: _________Years old

3. Major: A. Medical B. Nursing C. Logistics

4. Education: A. Diploma B. Bachelors Degree C. Master Degree

5. Years of work: _________Years

6. Professional title: A. Junior B. Intermediate C. Senior

7. Have you received any TCCC training? A. Yes B. No

8. Have you ever participated in military medical exercises? A. Yes B. No

**Part II Knowledge Test (25 questions in total)**

1. Which of the following is not one of the three preventable causes of death on the battlefield?

A. Massive bleeding B. traumatic brain injury C. airway obstruction D. Tension pneumothorax

2. What is the preferred rescue measure for tension pneumothorax?

A. External chest compressions B. thoracic puncture decompression C. Initiating an AED

3. What is the location of thoracic puncture decompression?

A. The 2nd intercostal medial clavicular line on the affected side

B. The 4th intercostal medial clavicular line on the affected side

C. The 2nd intercostal anterior axillary line on the affected side

D. The 4th intercostal anterior axillary line on the affected side

4. What is the angle of thoracic puncture decompression?

A. 45 degrees B. 60 degrees C.90 degrees

5. What is the depth of thoracic puncture decompression?

A. 3-5cm B. 5-8cm C. 8-10cm

6. For patients with hemorrhagic shock, what is the priority of resuscitation fluid?

A. ① Lactate Ringer's solution; ② hydroxyethyl starch; ③ Plasma; ④ The ratio of plasma, erythrocyte and platelet was 1:1:1; ⑤ Blood products; ⑥ Whole blood

B. ① Whole blood; ② The ratio of plasma, erythrocyte and platelet was 1:1:1; ③ Plasma; ④ Blood products; ⑤ hydroxyethyl starch; ⑥ Lactate Ringer's solution

C. ① Whole blood; ② The ratio of plasma, erythrocyte and platelet was 1:1:1; ③ Blood products; ④ Plasma; ⑤ hydroxyethyl starch; ⑥ Normal saline

D. ① Normal saline; ② hydroxyethyl starch; ③ Plasma; ④ The ratio of plasma, erythrocyte and platelet was 1:1:1; ⑤ Blood products; ⑥ Whole blood

7. A wounded with closed thigh fracture, clear consciousness, complaining of severe pain, has not established venous access, which of the following is the most appropriate analgesic method?

A. Taking anti-inflammatory painkiller orally

B. Appling fentanyl mucosal lozenges

C. 5mg Morphine by intramuscular injection

D. 50mg Ketamine by intramuscular injection

8. A wounded suffered from severe bleeding in both thighs. The bleeding had been stopped with a junction hemostat and venous access had been established. The wounded complained of severe pain and there was the possibility of continued shock and respiratory distress. What was the most appropriate way to relieve pain?

A. Intravenous/intraosseous injection of 5mg morphine

B. Intravenous/intraosseous injection of 10mg morphine

C. Intravenous/intraosseous injection of ketamine 20mg

D. Intravenous/intraosseous ketamine injection of 30mg slowly or intramuscular ketamine injection of 50mg

9. For the wounded who cannot take oral antibiotics, such as those in shock or coma, what is the appropriate antibiotic?

A. Take moxifloxacin 400mg orally

B. Intravenous/intramuscular injection of Ertapenem 1mg

C. Intravenous/intramuscular injection of Ertapenem 1g

D. Intravenous/intramuscular injection of Cefotetan 2mg

10. A wounded was shot in the abdomen during the firefight, blood gushing out, conscious, unbearable pain, at this time how to do?

A. Move him to the nearest shelter under cover of fire

B. Tamp his abdomen to stop bleeding under cover of fire

C. Give him analgesic and anti-inflammatory drugs under cover of fire

D. Encourage him to persist in fighting with a gun

11. Which order of assessment is the most appropriate in the tactical area treatment phase?

A. Consciousness → bleeding → airway → breathing → circulation → systemic examination (head to toe) → hypothermia prevention

B. Systemic examination (head to toe) → consciousness → bleeding → airway → respiration → circulation → hypothermia prevention

C. Consciousness → airway → breathing → circulation → bleeding → systemic examination (head to toe) → hypothermia prevention

D. Bleeding → circulation → consciousness → airway → respiration → systemic examination (head to toe) → hypothermia prevention

12. Which of the following combinations is not true?

A. Life-threatening severe bleeding of extremities: CAT tourniquet

B. Bleeding at junction: JETT junction forceps

C. Torso bleeding: tamponade pressure dressing to stop bleeding

D. Minor bleeding: pelvic fixation band

13. A wounded was shot in the left leg during the firefight, bleeding, blood soaked through the pants, which of the following is the most appropriate rescue at this time?

A. Tie A tourniquet at the root of the left thigh outside the military uniform

B. Tie two tourniquets at the root of the left thigh outside the military uniform

C. Cut open the military uniform, expose the wound, and apply a tourniquet near the heart of the wound on the skin

D. Cut the military uniform, expose the wound, and apply two tourniquets near the heart of the wound on the skin

14. During the treatment stage in the tactical area, when the wounded was examined by cutting open his uniform, blood was still pouring from the wound. At this time, which of the following is the most appropriate rescue?

A. Apply a tourniquet directly to the proximal skin without loosening the original tourniquet

B. Apply the tourniquet directly to the distal end of the skin without loosening the original tourniquet

C. Quickly loosen the original tourniquet and re-apply 2 tourniquets 5-8cm from the proximal end of the wound on the skin

D. Quickly release the tourniquet and stop the bleeding with a pressure dressing

15. A wounded was wounded by shrapnel in the chest, the right chest wall was punctured with fractured ribs, right chest wall collapsed, and can hear the air with breathing in and out of the "hiss - hiss" sound, the diameter of the wound is about 4cm, breathing difficulties, cyanosis. Which of the following rescue is the most appropriate at this time?

A. When inhaling, stick a sealing sticker with one-way vent hole

B. When exhaling, apply sealing tape with one-way vent hole

C. When inhaling, attach sealing tape without one-way vent hole

D. When exhaling, apply sealing tape without one-way vent hole

16. The wounded suffered a bullet wound in his right chest and his breathing difficulties were relieved after sealed with a sealant. However, 5 minutes later, he developed shortness of breath, cyanosis, right chest distension, reduced breathing amplitude, loss of breathing sound, and percussion symptoms of drum sound again. Which of the following measures is most appropriate at this time?

A. Remove one side of the seal sticker

B. Thoracic puncture decompression, right thoracic midclavicular line, 2nd intercostal

C. Thoracic puncture decompression, right thoracic midclavicular line, 4th intercostal line

D. Thoracic puncture decompression, right thoracic axillary line, 4th intercostal line

17. Which of the following is not part of a rapid vision test for the wounded with penetrating wound of eyes or corneal lacerations?

A. Test to read the printed material

B. Test to see if you can count how many fingers the paramedic holds up

C. Test to see the paramedic shaking his finger

D. To see a specific target 10 meters away

18. Which of the following is incorrect for the management of penetrating wound of eyes?

A. Complete a rapid vision test

B. Cover the injured eye with a stiff patch

C. Apply pressure patch to protect injured eyes

D. Take anti-inflammatory painkiller orally

19. Which of the following wounded most need to monitor oxygen saturation?

A. Open fracture of thigh B. Traumatic brain injury

C. 50% burn D. Abdominal penetrating wound

20. The patrol was attacked by an improvised explosive device. One wounded was unconscious, gasping for breath, unable to reach the carotid pulse. What was the most appropriate next action?

A. Perform CPR immediately B. Abandon rescue

C. Bilateral thoracic puncture decompression D. intravenous epinephrine 1mg

21. A wounded suffered facial burns, dyspnea, breathing 30 times per minute and rapid pulse. Which of the following measures is most appropriate at this time?

A. Insert nasopharyngeal vent tube B. Insert oropharyngeal vent tube

C. Incision of cricothyroid membrane D. Tracheal intubation

22. The wounded were burned on the head, face and trunk (front chest, back and perineum), with obvious edema, small blisters, thick blisters, and red and white wound base. The wounded weighed 80kg. Please calculate the burn area.

A. 27% B.30% C.33% D.36%

23. Which of the following is not true about the extent of the burn?

A. Minor burns: the burn area ofⅡdegree＜5%

B. Moderate burns: 10%-29% burn area of Ⅱdegree burns, or the burn area ofⅡdegree burns＜10%

C. Severe burns: 30%-50% of the total burn area, or 10%-20% burn area of the Ⅲ degree, or complicated with shock, respiratory tract burn or combined with severe compound injury

D. Extreme burn: total burn area > 50% or Ⅲ degree burn area > 20%, or severe complications

24. Unilateral pupil dilation with decreased level of consciousness indicates an impending cerebral hernia. Which of the following methods for reducing intracranial pressure is incorrect?

A. Intravenous drip 250ml hypertonic saline (3% or 5%)

B. Raise head 30 degrees

C. Hyperventilation: balloon mask connected to oxygen reservoir mask for ventilation

D. Respiratory rate 20 times/min

E. Maintain end-expiratory partial pressure of CO_2_ at 30-35mmHg

F. Set oxygen concentration to medium flow

25. Among the monitoring indexes of traumatic brain injury, what are the range of SBP, SPO_2_ and PaCO_2_ respectively?

A. SBP > 90mmHg, SPO_2_ > 90%, PaCO_2_ in the range of 35-40 mmHg

B. SBP > 100mmHg, SPO_2_ > 94%, PaCO_2_ in the range of 25-35 mmHg

C. SBP > 110mmHg, SPO_2_ > 96%, PaCO_2_ in the range of 15-25 mmHg

D. SBP > 120mmHg, SPO_2_ > 98%, PaCO_2_ in the range of 45-55mmHg
