# Supplementary material for: A Serious Game for Enhancing Rescue Reasoning Skills in Tactical Combat Casualty Care: Development and Deployment Study
Source: JMIR Form Res. 2024 Aug 12;8:e50817. doi: 10.2196/50817 (PMC11347892; doi:10.2196/50817)
Supplement: Multimedia Appendix 2 [file formative_v8i1e50817_app2.doc]

**Appendix 2**

**Self-confidence rating form**

| **Item** | **Before simulation training** | | | | | **After simulation training** | | | | |
| --- | --- | --- | --- | --- | --- | --- | --- | --- | --- | --- |
| 1. I have confidence in my basic knowledge | 1 | 2 | 3 | 4 | 5 | 1 | 2 | 3 | 4 | 5 |
| 2. I have faith in the latest ideas and research progress | 1 | 2 | 3 | 4 | 5 | 1 | 2 | 3 | 4 | 5 |
| 3. I have the confidence to assess the injury accurately | 1 | 2 | 3 | 4 | 5 | 1 | 2 | 3 | 4 | 5 |
| 4. I have the confidence to prioritize the injuries | 1 | 2 | 3 | 4 | 5 | 1 | 2 | 3 | 4 | 5 |
| 5. I have the confidence to discern the changes of injury independently | 1 | 2 | 3 | 4 | 5 | 1 | 2 | 3 | 4 | 5 |
| 6. I have the confidence to manage injuries independently | 1 | 2 | 3 | 4 | 5 | 1 | 2 | 3 | 4 | 5 |
| 7. I have the confidence to participate in military exercises | 1 | 2 | 3 | 4 | 5 | 1 | 2 | 3 | 4 | 5 |
| 8. I have the confidence to fulfill duties and missions of the mobile medical logistics teams | 1 | 2 | 3 | 4 | 5 | 1 | 2 | 3 | 4 | 5 |
