# Supplementary material for: A Serious Game for Enhancing Rescue Reasoning Skills in Tactical Combat Casualty Care: Development and Deployment Study
Source: JMIR Form Res. 2024 Aug 12;8:e50817. doi: 10.2196/50817 (PMC11347892; doi:10.2196/50817)
Supplement: Multimedia Appendix 3 [file formative_v8i1e50817_app3.doc]

**Satisfaction questionnaire**

| **Item** | **Strongly disagree** | **Disagree** | **Not sure** | **Agree** | **Strongly agree** |
| --- | --- | --- | --- | --- | --- |
| 1. Vivid software interface | 1 | 2 | 3 | 4 | 5 |
| 1. Easy to use | 1 | 2 | 3 | 4 | 5 |
| 1. Appropriately difficult injury | 1 | 2 | 3 | 4 | 5 |
| 1. Prompt, effective answers | 1 | 2 | 3 | 4 | 5 |
| 1. Aroused interest in learning TCCCa | 1 | 2 | 3 | 4 | 5 |
| 1. Improved basic TCCC knowledge | 1 | 2 | 3 | 4 | 5 |
| 1. Increased the importance of TCCC for me | 1 | 2 | 3 | 4 | 5 |
| 1. Improved my reasoning ability within TCCC | 1 | 2 | 3 | 4 | 5 |
| 1. Promoted the combined use of theory and practice | 1 | 2 | 3 | 4 | 5 |
| 1. Ensured the study of TCCC serves a practical purpose | 1 | 2 | 3 | 4 | 5 |

^a^TCCC: tactical combat casualty care
